# Gut microbiota mediates the anti-obesity effect of calorie restriction in mice

**DOI:** 10.1038/s41598-018-31353-1

**Published:** 2018-08-29

**Authors:** Shuo Wang, Meiqin Huang, Xue You, Jingyu Zhao, Lanlan Chen, Lin Wang, Yangjun Luo, Yan Chen

**Affiliations:** 1 CAS Key Laboratory of Nutrition, Metabolism and Food Safety, Shanghai Institute of Nutrition and Health, Shanghai Institutes for Biological Sciences, University of Chinese Academy of Sciences, Chinese Academy of Sciences, Shanghai, 200031 China; 2 School of Life Sciences and Technology, Shanghai Tech University, Shanghai, 200031 China

## Abstract

Calorie restriction (CR) extends lifespan and elicits numerous effects beneficial to health and metabolism in various model organisms, but the underlying mechanisms are not completely understood. Gut microbiota has been reported to be associated with the beneficial effects of CR; however, it is unknown whether these effects of CR are causally mediated by gut microbiota. In this study, we employed an antibiotic-induced microbiota-depleted mouse model to investigate the functional role of gut microbiota in CR. Depletion of gut microbiota rendered mice resistant to CR-induced loss of body weight, accompanied by the increase in fat mass, the reduction in lean mass and the decline in metabolic rate. Depletion of gut microbiota led to increases in fasting blood glucose and cholesterol levels independent of CR. A few metabolism-modulating hormones including leptin and insulin were altered by CR and/or gut microbiota depletion. In addition, CR altered the composition of gut microbiota with significant increases in major probiotic genera such as *Lactobacillus* and *Bifidobacterium*, together with the decrease of *Helicobacter*. In addition, we performed fecal microbiota transplantation in mice fed with high-fat diet. Mice with transferred microbiota from calorie-restricted mice resisted high fat diet-induced obesity and exhibited metabolic improvement such as alleviated hepatic lipid accumulation. Collectively, these data indicate that CR-induced metabolic improvement especially in body weight reduction is mediated by intestinal microbiota to a certain extent.

## Introduction

Calorie restriction (CR) is a dietary regimen that reduces calorie intake without incurring malnutrition. Countless researches since the 1930s have confirmed that CR is the only effective environmental intervention that is known to extend lifespan in many organisms including yeast, worms, flies, rodents and perhaps non-human primates^1–7^. Also, CR has been reported to prevent the occurrence of metabolic syndromes such as obesity and reduce the risk factors of age-associated diseases such as cancer, diabetes and atherosclerosis in many mammals, including humans^8–13^. However, the underlying mechanisms remain controversial.

It is widely accepted that metabolic syndromes and many age-associated disorders are intimately linked to diet. Furthermore, extensive research on gut microbiota represents clear evidence that diet modulates the composition and function of these microbes and the diet-microbiota interactions are pivotal moderators of metabolism^14–22^. Especially, emerging studies have demonstrated that gut microbiota can alter the absorption, metabolism and storage of calories^23–25^, although the actual mechanisms are difficult to elucidate. CR, as an important way of dietary intervention, could reshape the gut microbiota. Recent studies witnessed significant dynamic changes of mice and human gut microbiota as response to calorie-restricted diet^26,27^, although the overall bacterial phylogenetic alteration was not substantially affected by CR in humans^28^. However, it remains to be determined whether the phenomenon that CR alleviates metabolic syndromes is causally mediated by gut microbiota and if so, what are the underlying mechanisms. In this study, we used an antibiotic-induced microbiota-depleted (AIMD) mouse model combined with fecal microbiota transplantation (FMT) to investigate whether or not gut microbiota is causally involved in metabolic improvement associated with calorie restriction.

## Results

### Antibiotic treatment extensively depletes commensal gut microbiota

**Figure 1:**
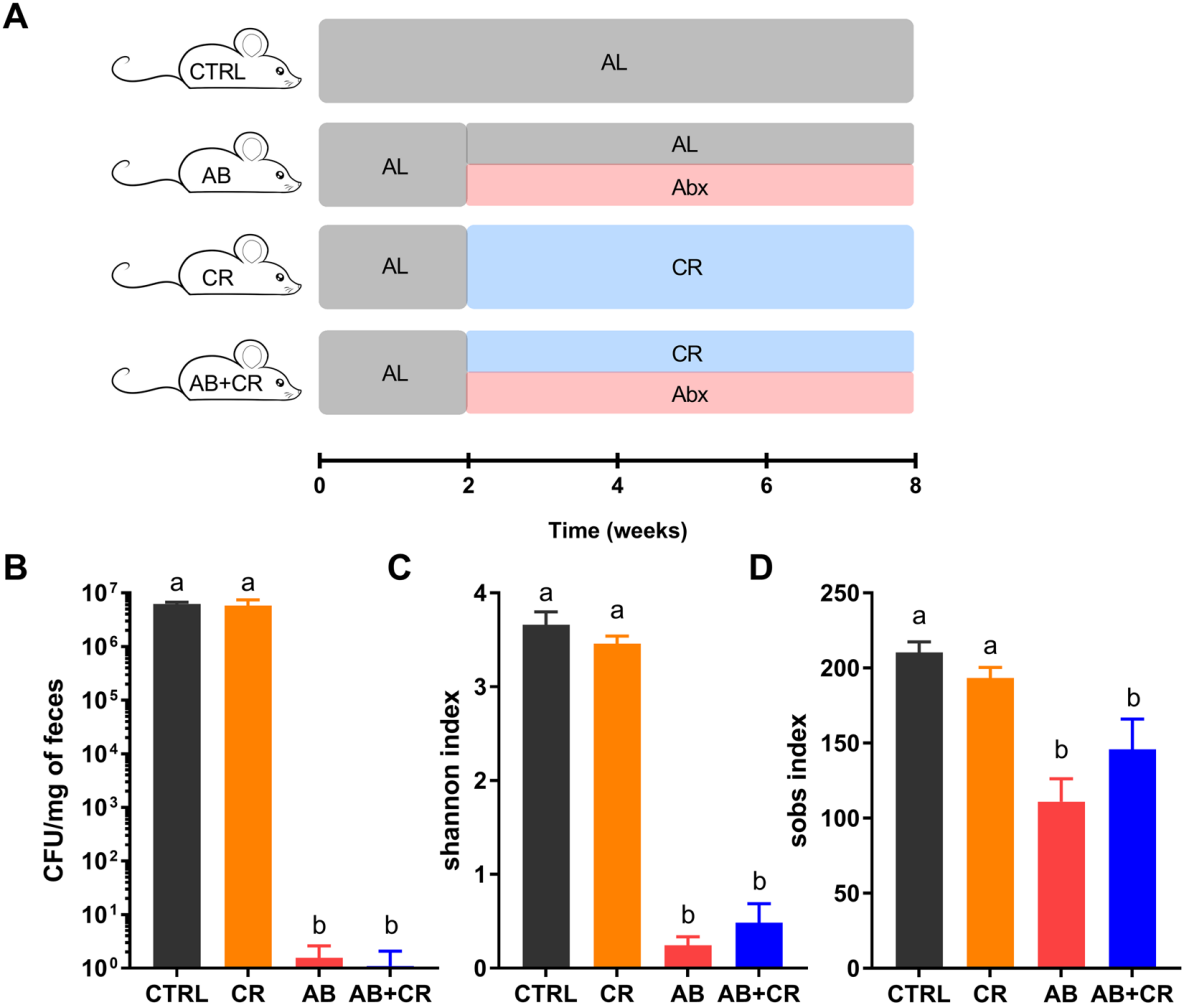
Depletion of gut microbiota after antibiotic treatment. (**A**) Schematic design of the experiment. AL, ad libitum; Abx, antibiotic treatment. (**B**) Fecal bacterial loads in all groups of mice. Experiments were repeated 3 times. (**C**,**D**) Effects of antibiotic treatment on diversity and richness of fecal microbiota revealed respectively by shannon index (C) and sobs index (D). Data are expressed as means ± s.e.m, n = 9–10 per group.

Following the schematic as described in Fig. 1A, we investigated four groups of mice in the experiment. In the control group (CTRL), the mice were continuously fed normal chow ad libitum. The calorie-restricted group (CR) was fed with a 70% of normal chow based on the food intake of CTRL group. Microbiota was depleted by antibiotic treatment in mice continuously fed ad libitum (AB) or fed with a 30% calorie-restriction diet (AB + CR). We started treating mice in AB and AB + CR groups with four nonabsorbable broad-spectrum antibiotics at the onset of calorie restriction. Fecal bacterial loads were examined by cultivation of anaerobic microbes using serial dilutions of resuspended fecal pellets on brain heart infusion (BHI) agar plates. Antibiotic treatment remarkably reduced culturable gut microbes by about one million-fold (Fig. 1B). Significant reduction of community diversity and richness of gut microbiota by the antibiotics were reflected by analyses of shannon index (Fig. 1C) and sobs index (Fig. 1D) via 16S rRNA gene sequencing, further indicating the effectiveness of antibiotic treatment.

### Microbiota-depleted mice are resistant to CR-induced body weight loss

**Figure 2:**
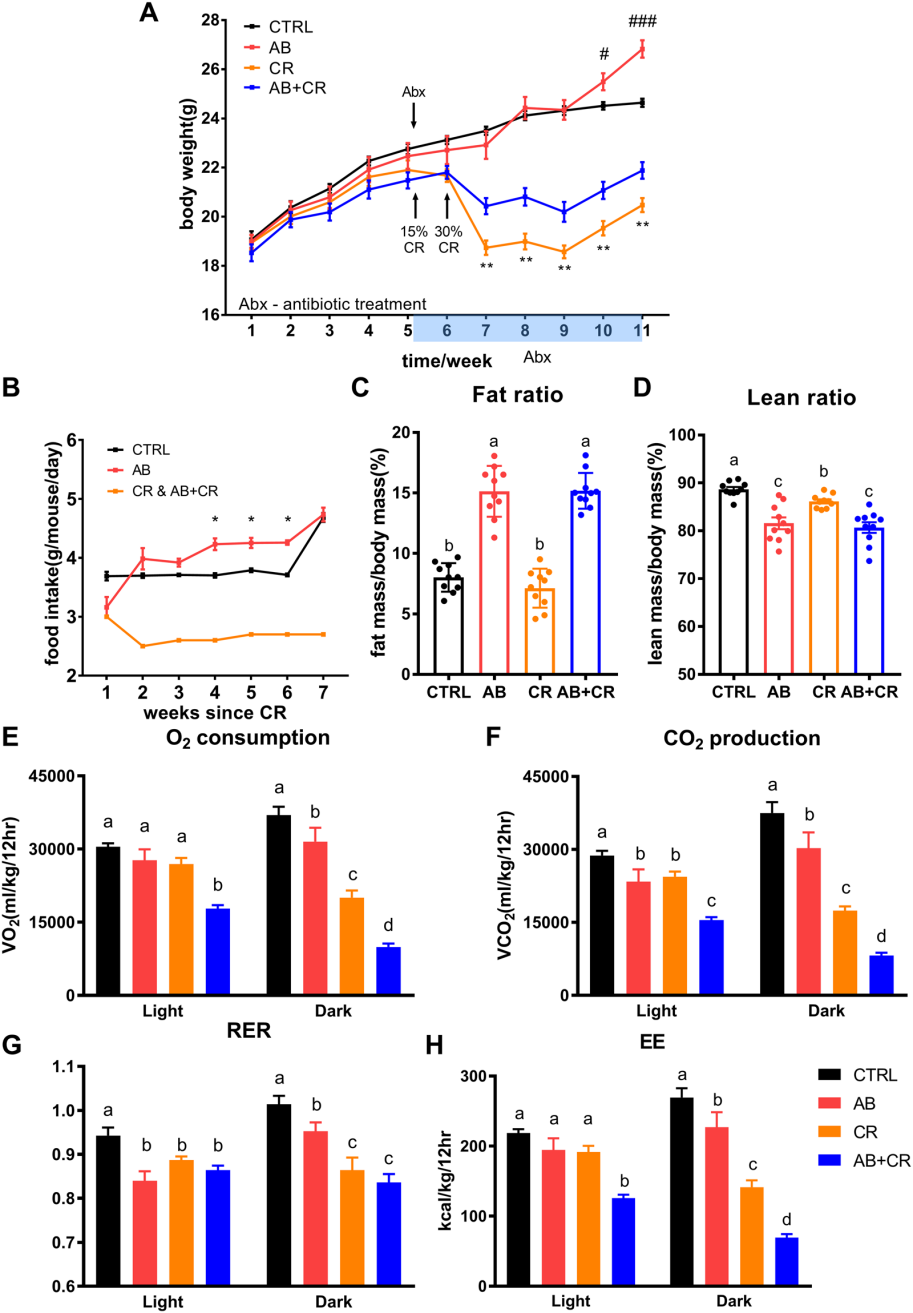
Body weight and metabolic alterations upon CR and gut microbiota depletion. (**A**) Body weight curves of the mice (n = 9–10 per group). **p < 0.01 between CR and AB + CR groups, ^#^p < 0.05 and ^###^p < 0.001 between CTRL and AB groups. (**B**) Food intake (n = 9–10 per group). *p < 0.05 between AB and CTRL groups. (**C**,**D**) Quantification of body fat mass (C) and lean mass (D) by MRI scans (n = 9–10 per group). (**E**–**H**) Analyses with metabolic chamber to quantitate O_2_ consumption (**E**), CO_2_ production (**F**), respiratory exchange ratio (RER) (**G**) and energy expenditure (EE) (**H**) (n = 4 for each group). Data are expressed as means ± s.e.m.

Upon antibiotic treatment and calorie restriction, the control mice and the microbiota-depleted mice responded differently to calorie restriction. As expected, CR was able to reduce body weight starting from the second week of application of CR diet (Fig. 2A). Since then, the mice without gut microbiota (AB + CR) experienced less body weight loss than the CR group (Fig. 2A), although the two groups of mice had equal levels of food intake (Fig. 2B). The significant discrepancy of body weight between the two groups stayed until the end of the experiment (Fig. 2A). In addition, at the end of the experiment, AB group gained more weight than the CTRL group (Fig. 2A), accompanied by an increase in food intake in the last few weeks (Fig. 2B). These results, therefore, indicated that gut microbiota plays an important role in CR-induced loss of body weight.

### Analyses of metabolic and blood parameters

We analyzed a few metabolic parameters of the mice. As compared to the mice without antibiotic treatment, microbiota-depleted mice were characterized by a significant increase in total body fat and a decrease in lean mass as determined by MRI (Fig. 2C,D). CR could slightly reduce the ratio of lean mass (Fig. 2D). However, the AB + CR group lost more lean mass than the CR group (Fig. 2D). In terms of metabolic rate or energy expenditure (EE) represented by O_2_ consumption, CO_2_ production and energy expenditure, the AB + CR group had the lowest metabolic rate among the four experimental groups, especially in the dark phase (Figs 2E–G and S1). In addition, CR was able to reduce metabolic rate during the dark period (Fig. 2E–G). Respiratory exchange ratio (RER) was reduced by either calorie restriction or antibiotic administration in light phase (Fig. 2H). In dark phase, calorie restriction was more effective to reduce RER in the mice treated with antibiotics (Fig. 2H). As decreased energy expenditure was commonly associated with weight gain and obesity^29,30^, our data suggested that the observed abrogation of CR-mediated body weight loss by microbiota deletion might be caused by a decrease in metabolic rate upon antibiotics administration. In other words, gut microbiota is likely important for the mice to maintain a relatively high level of metabolic rate so that depletion of the microbiota would result in a significant reduction of metabolic rate.

**Figure 3:**
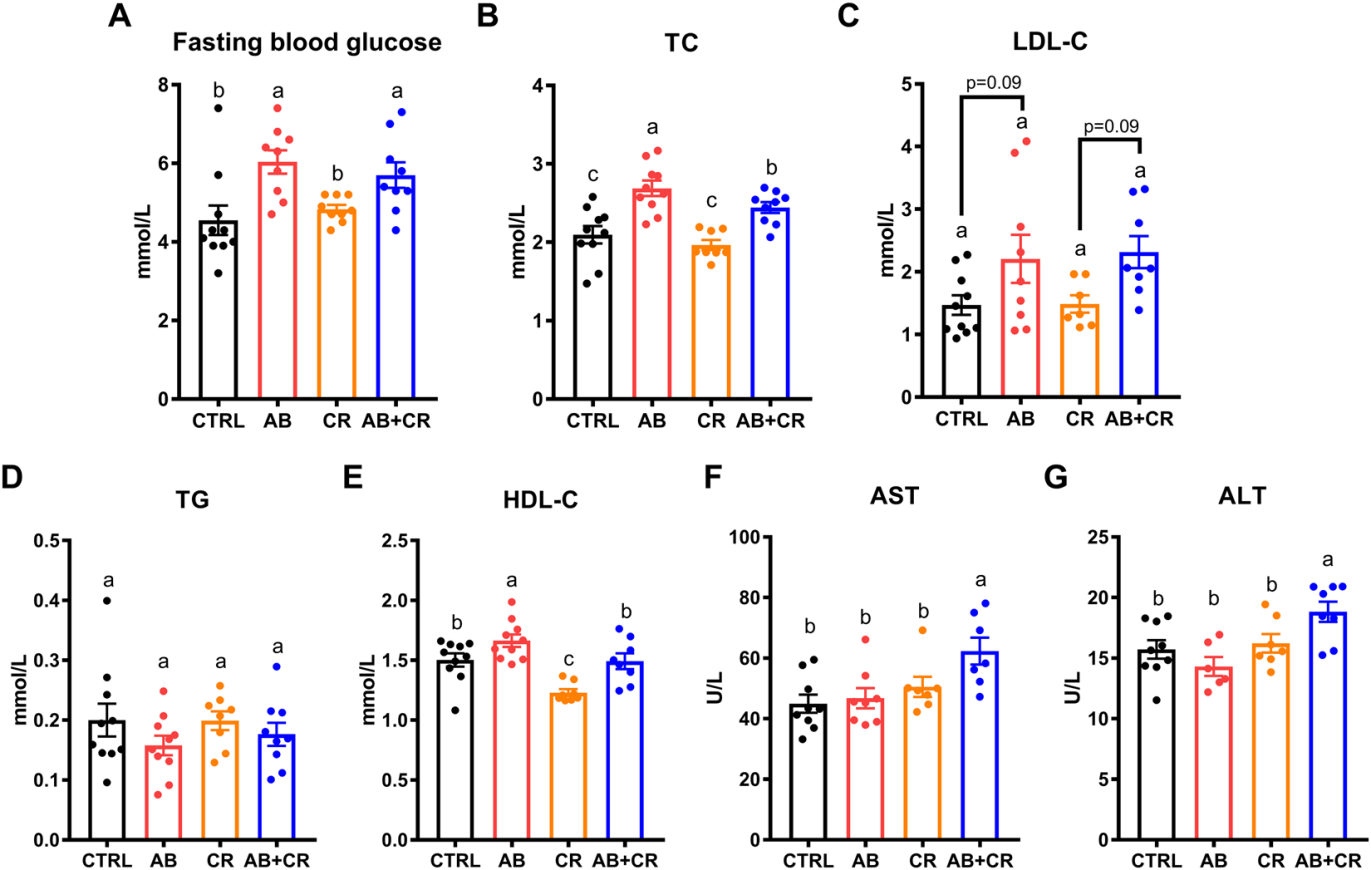
Impacts of CR and gut microbiota depletion on blood parameters. (**A**) Fasting blood glucose level in different groups of mice. (**B**–**G**) Blood levels of TC (**B**), LDL-C (**C**), TG (**D**), HDL-C (**E**), AST (**F**) and ALT (**G**) of the mice at the end of the experiment. Data are expressed as means ± s.e.m, n = 9–10 per group.

We also determined the fasting blood glucose and plasma levels of triglycerides (TG), total cholesterol (TC), high-density lipoprotein cholesterol (HDL-C) and low-density lipoprotein cholesterol (LDL-C). Antibiotic treatment elevated fasting blood glucose level and plasma TC level in the mice (Fig. 3A,B), with mild alteration on plasma LDL-C level (Fig. 3C). These data indicated that gut microbiota depletion is able to increase the risk of metabolic dysregulation as the elevations of blood glucose and cholesterol levels are considered as the hallmarks of metabolic syndrome^31,32^. On the other hand, the four groups of mice had minimal change in plasma TG and HDL-C (Fig. 3D,E). In addition, the AB + CR mice had a significant elevation of aspartate aminotransferase (AST) and alanine aminotransferase (ALT) as compared to other groups (Fig. 3F,G), indicating that liver damage might occur in response to calorie restriction in the absence of gut microbiota.

We also analyzed the histological and morphological changes of the mice. Antibiotic-treated mice were characterized by a significant increase in the length of the small intestine (Fig. S2A) and decreased liver/body weight ratio (Fig. S2B) but without apparent changes in the histology of the liver (figure not shown). Furthermore, under microscopical examination, hematoxylin-eosin staining of the jejunum sections in the antibiotic-treated mice revealed longer and thinner intestinal villi than the mice without antibiotic treatment (Fig. S2C).

### Gut microbiota depletion and calorie restriction alters metabolism-modulating hormones

**Figure 4:**
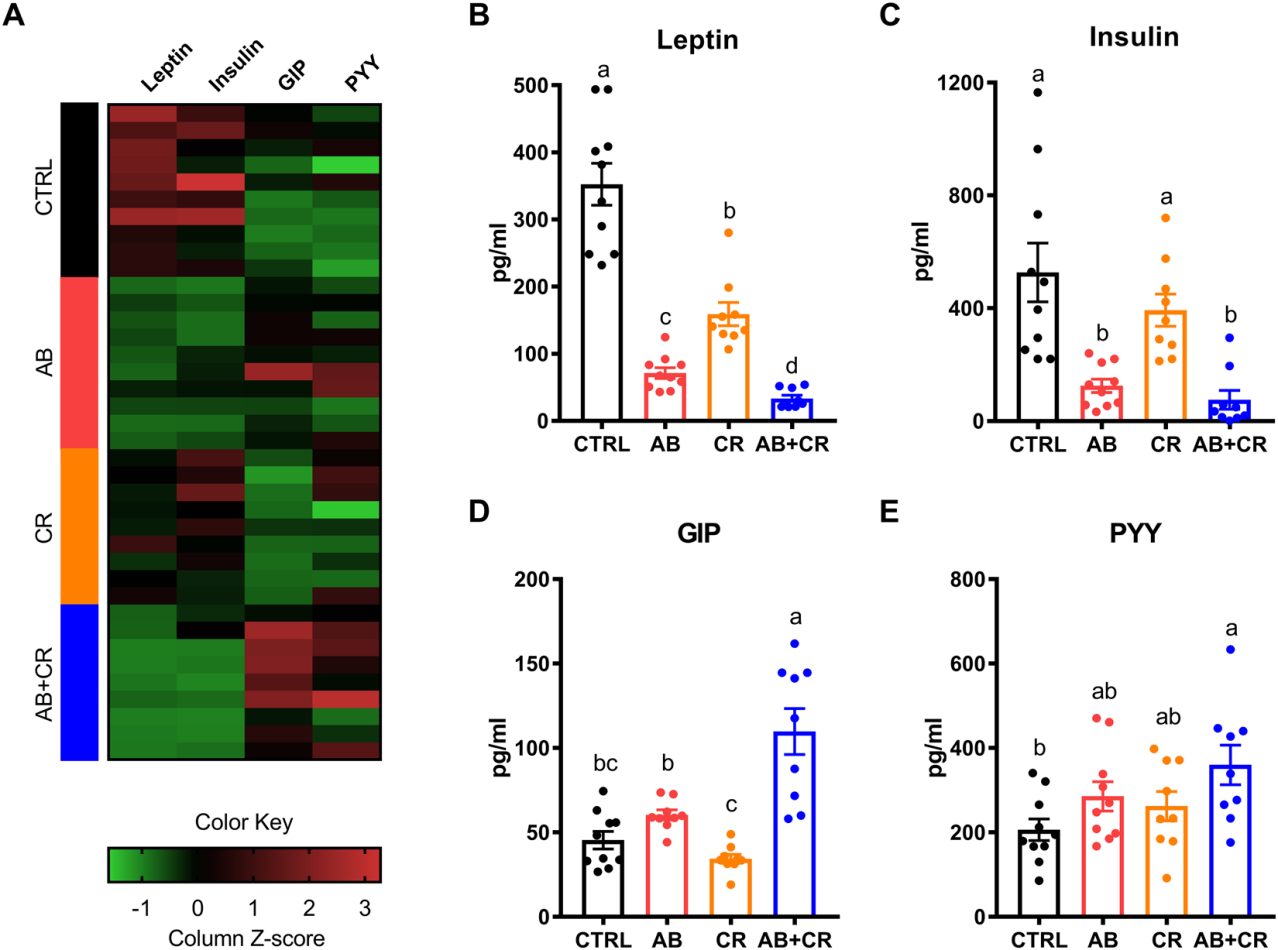
Role of CR and gut microbiota depletion on metabolic hormones. (**A**) Changes in plasma levels of related hormones shown by heatmap. (**B**–**E**) Blood levels of leptin (**B**), insulin (**C**), GIP (**D**) and PYY (**E**). Data are expressed as means ± s.e.m, n = 9–10 per group.

Interestingly, AB mice exhibited hyperphagia characterized by a significant increase in food intake (Fig. 2B). We thus assumed that the gut microbiota might be involved in the secretion of hormones that regulate body weight and appetite^33,34^. To verify our hypothesis, we determined the plasma levels of insulin, leptin, gastric inhibitory polypeptide (GIP) and peptide YY (PYY). The overall levels of these four hormones in all four groups were visualized by a heatmap shown in Fig. 4A. Leptin is a hormone that helps to regulate energy balance by suppressing hunger^35^ and leptin deficiency is commonly associated with obesity^36^. Calorie restriction could lower the plasma level of leptin, the effect of which was enhanced when the mice were exposed to antibiotic treatment (Fig. 4B). Insulin is known to regulate the metabolism of carbohydrates, lipids and proteins by aiding the body to store the glucose. Notably, similar to leptin, insulin is also an acute appetite suppressant^37^. Antibiotic treatment significantly decreased the plasma insulin level (Fig. 4C), consistent with the observed elevation of fasting blood glucose level upon antibiotics administration (Fig. 3A). GIP belongs to the family of incretins that are released by nutrients from the gastrointestinal tract to amply insulin secretion^38^. Besides its effects to induce insulin secretion upon glucose administration and to regulate fatty acid metabolism, GIP was recently found to be an obesity-promoting factor by acting on adipocytes^39,40^. Intriguingly, AB + CR mice had the highest level of GIP level among the four groups of mice (Fig. 4D). PYY is a hormone produced in the small intestine and helps to reduce appetite and limit food intake^41^. The plasma level of PYY in the AB + CR group was significantly higher than that of the CTRL group (Fig. 4E). Collectively, these data indicated that gut microbiota plays an important role in modulating hormones that regulate metabolism in the mice.

### Calorie restriction alters the composition of gut microbiota

**Figure 5:**
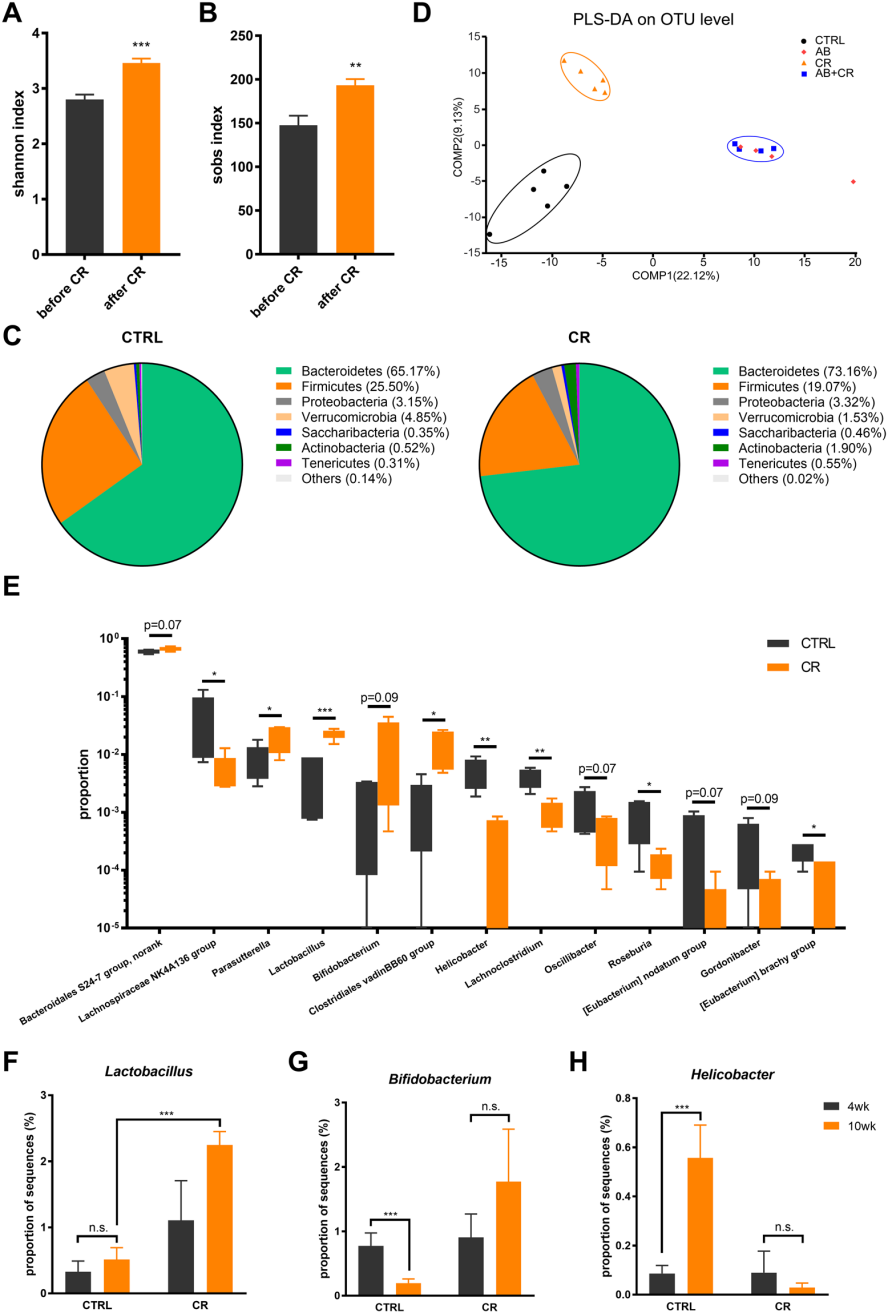
Structural rearrangement of gut microbiota in calorie-restricted mice. (**A**,**B**) Shannon index (A) and sobs index (B) of gut microbes as response to CR. (**C**) Composition of gut microbiota in CTRL and CR groups as shown in pie chart. (**D**) Partial least squares discriminant analysis (PLS-DA) of all the groups. (**E**) Generic differences between CTRL and CR groups with p-value < 0.1 in order of abundance. (**F**–**H**) Generic differences in *Lactobacillus* (**F**), *Bifidobacterium* (**G**) and *Helicobacter* (**H**) in the CTRL and CR groups at the 4th and 10th week of the experiment. Data are expressed as means ± s.e.m, n = 4–5 per group. *p < 0.05, **p < 0.01, ***p < 0.001.

We then performed 16S rRNA gene sequencing with the mice feces collected before and after administration of calorie restriction and/or antibiotic treatment, aiming to find how gut microbiota responds to calorie restriction and mediates the changes of body weight and metabolism. By significance tests for differences in α diversity, the gut microbiota of CR mice featured a markedly increased shannon index (Fig. 5A) and sobs index (Fig. 5B). As shown by respective rarefaction curves (Fig. S3), these curves became much flatter to the right, indicating that a reasonable number of sequences were taken and the α diversity of the sampled community was sufficiently extrapolated. Collectively, these data indicate that calorie restriction could render the gut microbiota a more balanced and diversified ecosystem.

The calorie restriction-induced difference in microbiota composition was illustrated in Fig. 5C. After calorie restriction, the bacterial phyla *Bacteroidetes* and *Actinobacteria* were slightly increased, while *Firmicutes* and *Verrucomicrobia* were slightly decreased. Also, a structural rearrangement of gut microbiota occurred after calorie restriction as illustrated by a supervised partial least squares discriminant analysis (PLS-DA) and hierarchical clustering (Figs 5D and S4). Notably, the antibiotic-treated mice exhibited no significant structural modulation under a calorie-restricted diet (Fig. 5D). Actually, based on the analysis of β diversity distance matrix, the bacterial structures in AB and AB + CR groups were more alike to each other after the experimental treatment (Fig. S5), as antibiotics extensively depleted resident gut microbiota.

We next performed linear discriminant analysis (LDA) effect size (LEfSe) analysis and identified a few bacterial genera that were significantly different between CTRL and CR groups (Figs 5E and S6). *Lactobacillus* and *Bifidobacterium* are widely approved probiotic genera with extensive health-promoting and immunomodulatory properties^42–44^. Compared to the CTRL group, the proportion of *Lactobacillus* and *Bifidobacterium* was increased in the CR group (p < 0.05 and p = 0.09 respectively, Fig. 5F,G). *Helicobacter* is a bacterial genus living mostly in the upper gastrointestinal tract which was often considered to be infectious and pathogenic^45^. We found that *Helicobacter* genus was significantly reduced by calorie restriction (Fig. 5H). In addition, a few other genera were altered by calorie restriction such as *Lachnospiraceae NK4A136*, *Parasutterella*, *Clostridiales vadinBB60*, *Lachnoclostridium*, *Oscillibaster*, *Roseburia* and *Gordonibacter* (Figs 5E and S6). Together, these data suggested that the composition and architecture of gut microbiota are altered by calorie-restricted diet, likely contributing to the metabolic benefits of CR.

### Gut microbiota contribute to CR-mediated metabolic improvement

**Figure 6:**
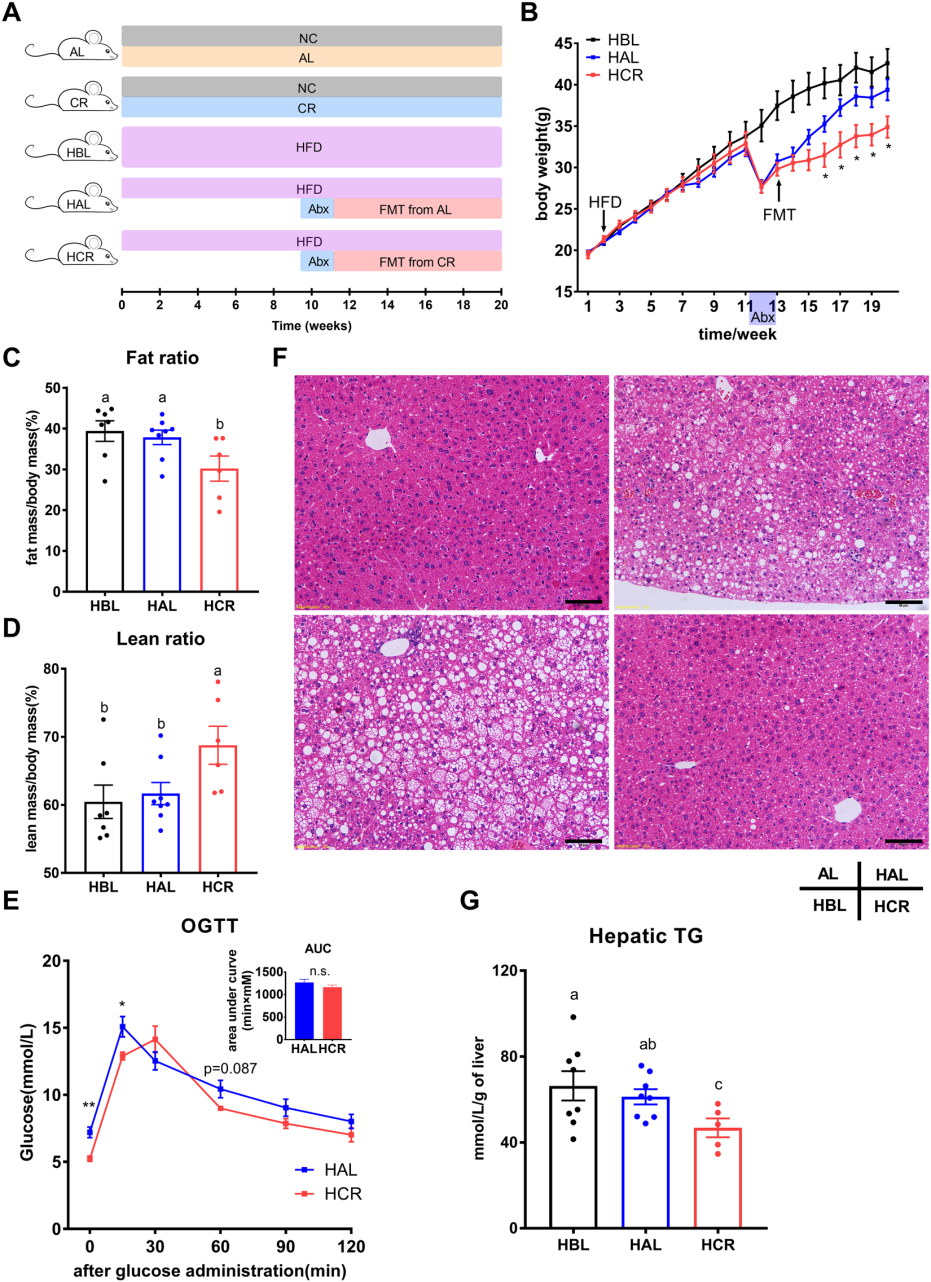
FMT from CR mice attenuated diet-induced obesity and partially ameliorated metabolic disturbances. (**A**) Schematic design of the FMT experiment. NC, normal chow; HFD, high fat diet. (**B**) Body weight curves of mice fed HFD (n = 6–8 per group). (**C**,**D**) Quantification of body fat mass (C) and lean mass (D) by MRI scans (n = 6–8 per group). (**E**) Oral glucose tolerance test of mice received FMT (n = 6–8 per group) with area under curve (AUC) shown in the inlet. (**F**) Representative H&E staining of the liver. Scale bar, 50 μm. (**G**) Hepatic triglyceride levels of the mice fed HFD (n = 6–8 per group). Data are expressed as means ± s.e.m, *p < 0.05, **p < 0.01.

To further investigate whether gut microbiota alteration during CR is causally associated with metabolic improvement of the mice, we performed fecal microbiota transplantation (FMT) in diet-induced obesity (DIO) mice using microbiota collected respectively from mice fed normal chow ad libitum (AL) or 30% CR (Fig. 6A). Upon transplantation, the microbiota of the recipient mice resembled their corresponding donor groups to certain degrees as evaluated by OTU level based on 16s rRNA gene sequencing (Fig. S7). Notably, compared to the mice undergoing FMT from the control AL mice (HAL group), the mice that received FMT from the CR mice (HCR group) exhibited reduced body weight gain (Fig. 6B), accompanied by a decrease in body fat mass and an increase in lean mass (Fig. 6C,D). In addition, there was a slight improvement in glucose tolerance together with a significant reduction of fasting blood glucose level in the HCR group as compared to the HAL group (Figs 6E and S8A). In addition, HCR mice exhibited a significant decrease in blood leptin level (Fig. S8B), which was largely consistent with our observation with the CR mice (Fig. 4B). We also determined the level of plasma insulin level but no significant difference was found among the three HFD groups (Fig. S8C). This result was also concordant with our previous data that plasma insulin level had minimal change between CTRL and CR groups (Fig. 4C). Besides, some other metabolic and blood parameters such as plasma AST, ALT, TG, TC, HDL-C, LDL-C and hepatic TC, showed no significant changes among these groups of mice (Fig. S9). Furthermore, we examined histological changes of the liver by hematoxylin-eosin staining. As compared to the HBL group, mice in the HAL group exhibited similar morphology shown as a similar degree of hepatosteatosis induced by HFD (Fig. 6F). However, the HFD-induced hepatic steatosis appeared to be significantly alleviated in the HCR group (Fig. 6F). Consistently, the hepatic triglyceride level of the HCR group was significantly lower than those of HBL and HAL groups (Fig. 6G).

**Figure 7:**
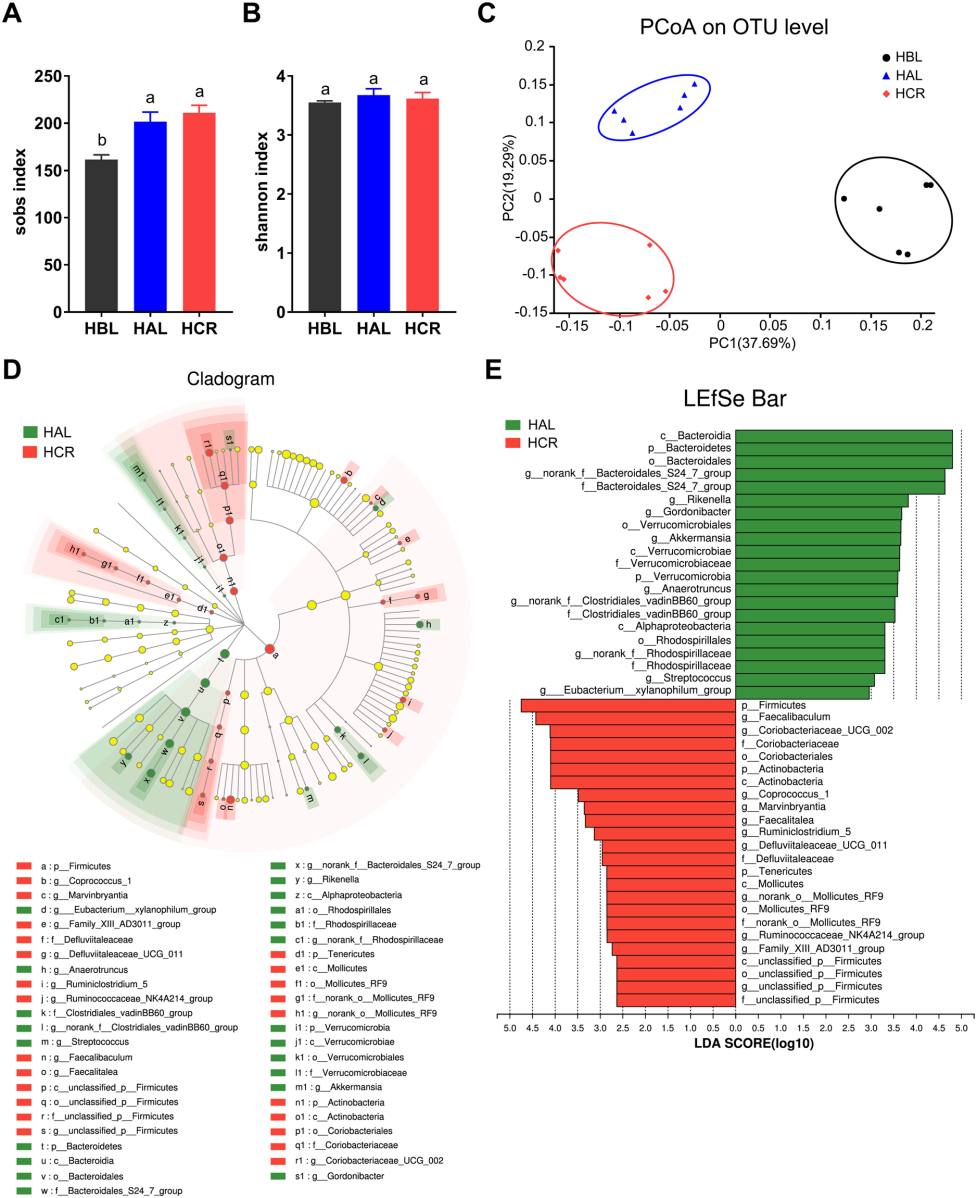
The impacts of FMT from CR mice on the gut microbiota of DIO mice. (**A**,**B**) Shannon index (A) and sobs index (A) of fecal microbiota in DIO mice. (**C**) Principal co-ordinates analysis (PCoA) of the groups fed HFD. (**D**,**E**) Linear discriminant analysis (LDA) effect size (LEfSe) analysis (D) and corresponding LDA scores (E) in the HAL and HCR groups. p, phylum; c, class; o, order; f, family; g, genus. Data are expressed as means ± s.e.m, n = 6 for each group.

We also analyzed the microbial composition of the three groups of mice fed with HFD. Compared to the HBL group, FMT elevated the richness of microbiota reflected as sobs index (Fig. 7A). The diversity of microbial community shown by shannon index among the three groups had minimal changes (Fig. 7B). However, the HAL and HCR groups did have a structural discrepancy in the gut microbiota as illustrated by principal co-ordinates analysis (PCoA) and hierarchical clustering (Figs 7C and S10). We also applied LEfSe analysis and identified a few altered bacteria between the HAL and HCR groups (Fig. 7D,E). Compared to the HAL group, the microbial composition in the HCR group had increases in the abundances of *Firmicutes* (59.75% vs. 71.68%, p < 0.05) and *Actinobacteria* (1.25% vs. 4.13%, p = 0.06), together with a decrease in the abundance of *Bacteroidetes* (35.35% vs. 21.46%, p < 0.05). At genus level, the HCR group had a significant increase in genus *Faecalibaculum*, which significantly contributed to the overall differences (Fig. 7E). Besides, abundances of some other genera were different between the HAL and HCR groups such as *Bacteroidales S24-7*, *Gordonibacter*, *Rikenella*, *Coriobacteriaceae UCG-002* and *Coprococcus 1* (Figs. 7D,E).

## Discussion

In our study, we aimed to investigate the causal role of gut microbiota in the modulatory effects of calorie-restriction diet on metabolism. We hypothesized that reduced calorie intake could induce structural and functional changes in gut microbiota, which further impacts the physiological and metabolic profiles and determines the effect of calorie restriction. Our study reveals that antibiotic-induced gut microbiota depletion abrogates the body weight-lowering and metabolism-benefitting effects of calorie restriction. Furthermore, the FMT experiment clearly indicates that the gut microbiota from the CR mice is able to alleviate obesity in DIO mice. These data thus indicate for the first time that gut microbiota plays a causal role to mediate the beneficial effects of calorie restriction. Therefore, the gut microbiota plays an important role to ensure that calorie restriction can bring about various beneficial activities such as lowering body weight and maintaining a high level of basal metabolic rate, decreasing blood glucose level and reducing serum cholesterol level. Antibiotic treatment, therefore, largely abolishes the metabolism-regulatory functions of gut microbiota and abrogates the health-beneficial effect of gut microbiota.

Our study also suggests that gut microbiota is required to maintain a certain degree of basal metabolic rate (BMR). The gut microbiota could protect against metabolic disturbances especially by preventing a fast decline of BMR. Previous studies have demonstrated a phenomenon of “metabolic adaptation” or “adaptive thermogenesis” in which the obese people exhibit lower reduction in BMR than expectation after weight loss^46–48^. It was also believed that this phenomenon may predispose people to post-dieting weight regain^49^. Our study reveals that the microbiota-depleted and calorie-restricted mice had the lowest metabolic rate among all the experimental groups (Fig. 2). Thus, depletion of gut microbiota may lead to loss of the “metabolic buffering” function of the gut microbiota, leading to abrogation of CR-mediated body weight loss. In addition to lowering metabolic rate to compensate for calorie deficiency upon CR, adapting energy loss through fecal calories was also observed in mouse studies^50^. The alteration of the nutrient load induced rapid changes in the gut microbiota and correlated with stool energy loss likely through alteration of the proportion of *Firmicutes* and *Bacteroidetes*^51^. However, in our experiment, none of *Firmicutes* genus, *Bacteroidetes* genus, nor *Firmicutes*/*Bacteroidetes* ratio was significantly changed by CR (data not shown).

Our study also indicates that gut microbiota is required to maintain the morphology of the small intestine. Depletion of gut microbiota was able to increase the length of the small intestine together with elongated intestinal villi (Fig. S2). Interestingly, other studies have revealed that calorie restriction could promote the preservation and self-renewal of intestinal stem cells with coordination of Paneth cells through reducing mTORC1 signaling^52^. A longer small intestine length and extension of intestinal villi upon microbiota depletion would provide a greater absorptive area, leading to an increase of energy intake. Such antibiotic-mediated changes in the morphology of small intestine may partly explain our observation that CR-induced body weight loss is abrogated by depletion of gut microbiota.

We found that gut microbiota is also required for regulating the secretion of metabolism-modulating hormones. It is now widely accepted that gut microbiota serves as an endocrine organ^53,54^ and a number of hormones are secreted directly by intestinal microbes, such as serotonin and dopamine^55,56^. Also, it has been found that many hormones secreted by the gastrointestinal tract itself and brain are indirectly regulated by gut microbiota possibly mediated by short chain fatty acids (SCFAs)^57^. We found that the blood levels of leptin, insulin, GIP and PYY were all altered by depletion of gut microbiota (Fig. 4). Therefore, our data would favor a model in which the gut microbiota plays an active role in regulating metabolism-modulating hormones secreted by the gastrointestinal tract and other tissues, so that antibiotic treatment would perturb secretion of these hormones and result in detrimental effects to the metabolic health.

Additionally, calorie restriction can also act on gut microbiota itself through adjustments of the diversity and the proportion of beneficial and harmful bacteria. Previous research reported that life-long calorie-restricted mice represent a structural modulation of gut microbiota and exert health benefits probably via reduction of antigen load from the gut^27^. In our study, the 6-week calorie restriction is adequate to elicit a few changes in gut microbiota. In addition to an increase in diversity, CR is able to increase probiotic genera such as *Lactobacillus* and *Bifidobacterium* and meanwhile reduce harmful bacteria such as *Helicobacter* (Fig. 6). We therefore propose that calorie restriction may bring about the beneficial effects to the body by reshaping the structure of gut microbiota.

In the FMT experiments, we found that the CR-mediated metabolic improvements were, at least partially, contributed by gut microbiota. The previous study had demonstrated that 8-week FMT intervention with donor mice fed ad libitum could attenuate HFD-induced steatohepatitis in mice^58^. Here we found that CR-induced microbiota alteration was effective to alleviate certain HFD-mediated metabolic aberrations such as diet-induced obesity and hepatic lipid accumulation (Fig. 6). Interestingly, FMT to HFD-fed mice from calorie-restricted animals led to an increase in *Faecalibaculum*, which significantly contributed to the overall differences (Fig. 7E). *Faecalibaculum*, which is featured by *F. rodentium*, was recently found to produce lactic acid as a major metabolic end product^59^. As lactic acid-producing bacteria were believed to possess an anti-obesity effect^60^, the increase of *Faecalibaculum* might partially contribute to the loss of body weight upon FMT from the CR mice.

In summary, our study demonstrates the key role of gut microbiota in mediating the effects of calorie restriction. Our results also suggest that gut microbiota dysbiosis caused by unfavorable dietary habit or environmental stimuli such as antibiotics usage may impose a risk to the metabolism of the body. In addition, gut microbiota may serve as a target in the prevention and treatment of metabolic disorders.

## Materials and Methods

### Mice

All animals were maintained and used in accordance with the guidelines of the Institutional Animal Care and Use Committee of the Institute for Nutritional Sciences, Chinese Academy of Sciences. All of the experimental procedures were carried out in accordance with the CAS ethics commission with an approval number 2010-AN-8. Male C57BL/6 mice at 4 wk of age, purchased from SLAC (Shanghai, China), were maintained in specific pathogen-free (SPF) conditions and kept on a 12 h light/dark cycle at the Institute for Nutritional Sciences. For our first animal experiment, all mice were weighed at the beginning and randomly allocated to one of the four groups so that the mean weights of each group were equal at the beginning of the experiment: (1) normal chow with free access to food and autoclaved water (CTRL, n = 10), (2) normal chow with 30% calorie restriction and autoclaved water (CR, n = 10), (3) normal chow with free access to food and antibiotic-treated water (AB, n = 11) and (4) normal chow with 30% calorie restriction and antibiotic-treated water (AB + CR, n = 10). For FMT experiment, all mice were weighed at the beginning and randomly allocated to one of the five groups: (1) normal chow ad libitum (AL, n = 5), (2) normal chow with 30% calorie restriction (CR, n = 5), (3) high-fat diet (Research Diet #D12492, with 60 kcal% fat) (HBL, n = 8), (4) high-fat diet undergoing FMT from AL group (HAL, n = 8) and (5) high-fat diet undergoing FMT from CR group (HCR, n = 7). All mice except for HFD mice were caged individually and maintained in sterile cages with autoclaved chip bedding. Mice fed HFD were caged 3–5 per cage.

### Calorie restriction

Before the start of calorie restriction and antibiotic treatment, all mice were allowed ad libitum access to food sterilized by ^60^Co irradiation from SLAC (Shanghai, China) and autoclaved water for acclimation and monitoring of daily food intake in the first 2 weeks. Calorie restriction was initiated with a 15% food reduction (3.1 g/day) for 5 days. Mice in CR and AB + CR groups were administered 70% of the food intake of CTRL group from 10 wk of age onward (2.5 g/day at first). Food was given at 13:00~15:00 p.m. each day to avoid disturbance of the circadian clock. The daily consumption of food in CTRL and AB groups was recorded every day before food administration. The food intake of CTRL group was averaged every week to determine the amount of food for the following week for the CR and AB + CR groups.

### Generation of antibiotic-induced microbiota-depleted (AIMD) mice

Antibiotic treatment was carried out together with calorie restriction. According to a previously published protocol^61^, 9-week old mice in AB and AB + CR groups received a combination of four nonabsorbable antibiotics: ampicillin, neomycin, metronidazole and vancomycin (Sangon Biotech, Shanghai, China) via oral gavage (0.2 mL) for 5 consecutive days (10 mg of each antibiotic per mouse per day) followed by administration in drinking water (ampicillin, neomycin and metronidazole: 1 g/L; vancomycin: 500 mg/L) which was renewed every week for the duration of the experiment. Mice in CTRL and CR groups received the same amount of sterile water by oral gavage in the first 5 days of treatment.

### Body/tissue measurement

Body weight of all mice was recorded weekly and on the day of sacrifice. The weights of liver and lengths of the small intestine were measured after dissection and liver/body weight ratios were calculated for each mouse.

### Mice fecal samples collection

Fresh fecal samples of all mice were collected at 15:00 ~ 17:00 p.m. to minimize possible circadian effects. Samples were collected into empty microtubes on ice and stored at -80 °C within 1 h for future use.

### Cultivation of anaerobic fecal microbiota

Freshly collected fecal pellets were weighed and suspended in 0.9% NaCl (10 mg/mL). After homogenization with a tissue grinder, the suspensions were performed a 10-fold serial dilution and plated on brain heart infusion (BHI) agar (Hopebio, China). All plates were incubated at 37 °C for 48 ~ 72 hours in anaerobic culture bags (Hopebio, China) with oxygen-reducing, carbon dioxide-generating sachets (Oxoid, Thermo Fisher Scientific, UK) and anaerobic indicators (Mitsubishi Gas Chemical, Japan). The number of CFU was counted and only anaerobic culturable fecal microbes could be quantified by this method.

### Body composition analysis

Mice body composition was assessed at 12 wk of age by echoMRI (Houston, USA) and the data of total body fat mass, lean mass, per cent body fat and per cent body lean were collected for each mouse, according to manufacturer’s directions.

### Measurement of metabolic rate and physical activity

Mice at 12 wk of age were randomly chosen (n = 4 for each group) for determination of metabolic rate using the comprehensive laboratory animal monitoring system (CLAMS-16, Columbus Instruments, USA) according to the manufacturer’s instructions. Mice were allowed to acclimate to the system for the first 24 h. Oxygen uptake (VO_2_), carbon dioxide production (VCO_2_) and respiratory exchange ratio (RER) were recorded in the following 24 h. All parameters above were collected with every period of 16 min.

### Measurement of plasma and liver parameters

Mice were euthanized and blood was immediately collected from the heart into EDTA-K2-treated microtubes (Kangjian Medical, China). Then the microtubes were centrifuged at 3,000 rpm for 15 min and the supernatant plasma was divided into 3 portions for different uses. All plasma samples excluding those for immediate uses were stored at −80 °C and multiple (>2) freeze/thaw cycles were avoided. Hepatic lipids were extracted with a previously reported method^62^. Plasma levels of aspartate transaminase (AST) and alanine transaminase (ALT) were determined by ALT/AST Determination Kit (ShenSuo UNF, China). Plasma and hepatic levels of triglycerides (TG), total cholesterol (TC), high-density lipoprotein cholesterol (HDL-C) and low-density lipoprotein cholesterol (LDL-C) were determined by the colorimetric method with corresponding kits (ShenSuo UNF, China) according to manufacturer’s instructions.

### Measurement of plasma hormones

Mice metabolic hormones were determined using either specialized ELISA kit or Milliplex MAP Magnetic Bead Mouse Metabolic Hormone Panel (Millipore MMHMAG-44K, Merck, Germany). Plasma leptin and insulin were determined respectively by mouse leptin ELISA Kit (MultiSciences, China) and mouse insulin ELISA Kit (Beyotime, China) according to manufacturer’s instructions. The Milliplex panel was custom-designed for the simultaneous quantification of GIP (total), insulin, leptin and PYY (total). Plasma samples (10 μL) were assayed using a Luminex MAGPIX analyzer (Merck Millipore, Germany) and the data were processed by Milliplex Analyst 5.1 software and R v3.3.2 (R Core Team, 2016).

### Hematoxylin-eosin staining of liver and small intestine samples

Mice livers and small intestines were dissected and washed in PBS. Liver and small intestine samples were fixed in 4% polyformaldehyde for 48 h and embedded in paraffin. Paraffin-embedded sections (4 μm) were subjected to standard hematoxylin-eosin staining.

### Fecal microbiota transplantation

Before FMT, mice were given a combination of ampicillin, neomycin, metronidazole and vancomycin (Sangon Biotech, China) in drinking water (ampicillin, neomycin and metronidazole: 1 g/L; vancomycin: 500 mg/L) for 10 consecutive days to remove indigenous gut microorganisms. After a 3-day recovery, FMT was operated twice a week. In brief, 200–300 mg of fresh stool was collected respectively from AL and CR group and was homogenated in 5 ml of PBS, settled by gravity for 2 min and the supernatant was gavaged 200 μl to each recipient mouse.

### Oral glucose tolerance test (OGTT)

Mice were fasted overnight and subsequently given 2 g/kg of glucose solution (Sigma-Aldrich, USA) by oral gavage. Blood glucose was determined at 0, 15, 30, 60, 90 and 120 min after glucose administration (Abbott, USA).

### Fecal DNA extraction, PCR amplification and Illumina MiSeq sequencing

Fecal DNA was extracted using the E.Z.N.A. stool DNA Kit (Omega Bio-tek, USA) according to the manufacturer’s instructions. DNA concentration was determined by a NanoDrop 2000 (Thermo Scientific, USA) and the integrity was examined by 1% agarose gel electrophoresis. The V3-V4 region of the bacterial 16S ribosomal RNA gene was amplified using primers 338 F (5′-ACTCCTACGGGAGGCAGCAG-3′) and 806 R (5′-GGACTACHVGGGTWTCTAAT-3′). The primers were incorporated with an 8-mer barcode sequence at the 5′ end which was unique to each sample. For PCR assays, 4 μL of 5 × Fast Pfu Buffer, 2 μL of 2.5 mM dNTPs, 0.8 μL of each primer (5 μΜ), 0.4 μL of FastPfu Polymerase (TransGen, China), 0.2 μL of BSA, 10 ng of template DNA and ddH_2_O were mixed to 20 μL in total. All PCR amplifications were performed in a thermocycler PCR system (ABI GeneAmp 9700, USA) in triplicate and following these steps: 95 °C for 3 min, followed by 30 cycles each consisting of 95 °C for 30 s, 55 °C for 30 s and 72 °C for 45 s, followed by a final extension of 72 °C for 10 min. Amplicons were examined and extracted from 2% agarose gels and purified using the AxyPrep DNA Gel Extraction Kit (Axygen Biosciences, USA) according to the manufacturer’s instructions and quantified using QuantiFluor-ST (Promega, USA). The purified amplicons from different samples were mixed at equal molar ratios and ligated with 300-bp paired-end adapters by TruSeq DNA Sample Prep Kit (Illumina, USA) then sequenced on an Illumina MiSeq platform (Illumina, USA) according to standard protocols.

### Bioinformatics and statistical analysis of sequencing data

The raw sequencing data were analyzed on the free online platform of Majorbio I-Sanger Cloud Platform (www.i-sanger.com, Majorbio, China). The raw paired-end reads were quality trimmed and filtered using Trimmomatic v0.32 (http://www.usadellab.org/cms/?page=trimmomatic)^63^. Then the optimized reads were merged with FLASH v1.2.11 (http://ccb.jhu.edu/software/FLASH/)^64^ according to their overlap sequences. The merged reads were divided by samples based on the barcode sequences. The average number of reads per sample was over 37,000 and the average length per read was 442 bp in our first animal model. In the FMT experiments, the average number of reads per sample was over 57,000 and the average length per read was 437 bp. After dereplication (http://drive5.com/usearch/manual/dereplication.html) and discarding the singletons (http://drive5.com/usearch/manual/singletons.html), the sequences were clustered into operational taxonomic units (OTUs) at 97% similarity using UPARSE v7.1 (http://drive5.com/uparse/) in QIIME (Quantitative Insights Into Microbial Ecology, http://www.qiime.org)^65^ while the chimeric sequences were identified and removed using UCHIME. The taxonomical classification was performed using RDP-classifier v2.2 (http://sourceforge.net/projects/rdp-classifier/) based on the SILVA 16S rRNA database (Release 128, http://www.arb-silva.de, Max Planck Institute, Germany) with a confidence threshold of 70%. Rarefaction curves and alpha diversity (Shannon) were determined using mothur v1.30.1 (http://www.mothur.org/wiki/Schloss_SOP#Alpha_diversity) and beta diversity was determined using QIIME. Unweighted UniFrac distance matrix analysis was performed in FastUniFrac (http://UniFrac.colorado.edu/) using OTUs for each sample. Partial least squares discriminant analysis (PLS-DA) is a supervised pattern recognition method and was achieved in R tools using package mixOmics. Significant changes in the abundance of OTUs or other levels between two groups were assessed using *Student’s* t test with bonferroni correction for multiple tests (α = 0.05). Linear discriminant analysis (LDA) coupled with effect size (LEfSe) was performed using LEfSe program (http://huttenhower.sph.harvard.edu/galaxy/root?tool_id = lefse_upload). A phylogenetic tree was built using FastTree v2.1.3 (http://www.microbesonline.org/fasttree/). All the graphs of bioinformatics unless otherwise stated, were generated by R v3.3.2 (R Core Team, 2016).

### Statistical analysis

Data are expressed as mean ± s.e.m. The unpaired *Student’s* t test with two tails was used to determine the significance of the differences between two groups. For data that showed a normal distribution and homogeneity of variance, a one-way ANOVA was performed for comparisons among more than two groups using an FDR *post hoc* analysis. To determine if two different factors have an effect on a measured variable, a two-way ANOVA was used, followed by FDR *post hoc* test. Comparisons of medians between non-normally distributed groups were performed using the Kruskal-Wallis H test for simultaneous comparisons of more than two groups. Statistical tests were performed using Microsoft Excel (Microsoft, USA), R v3.3.2 (R Core Team, 2016), or Prism 7 (GraphPad Software).

## Electronic supplementary material

Supplemental Figures
