# Supplementary material for: Gut microbiota mediates the anti-obesity effect of calorie restriction in mice
Source: Sci Rep. 2018 Aug 29;8:13037. doi: 10.1038/s41598-018-31353-1 (PMC6115465; doi:10.1038/s41598-018-31353-1)
Supplement: Supplementary file 1 — Supplemental Figures [file 41598_2018_31353_MOESM1_ESM.pdf]

Gut microbiota mediates the anti-obesity effect of calorie restriction in mice

Shuo Wang, Meiqin Huang, Xue You, Jingyu Zhao, Lanlan Chen, Lin Wang, Yangjun Luo, and Yan Chen

Supplemental Figures (There are 10 supplemental figures)

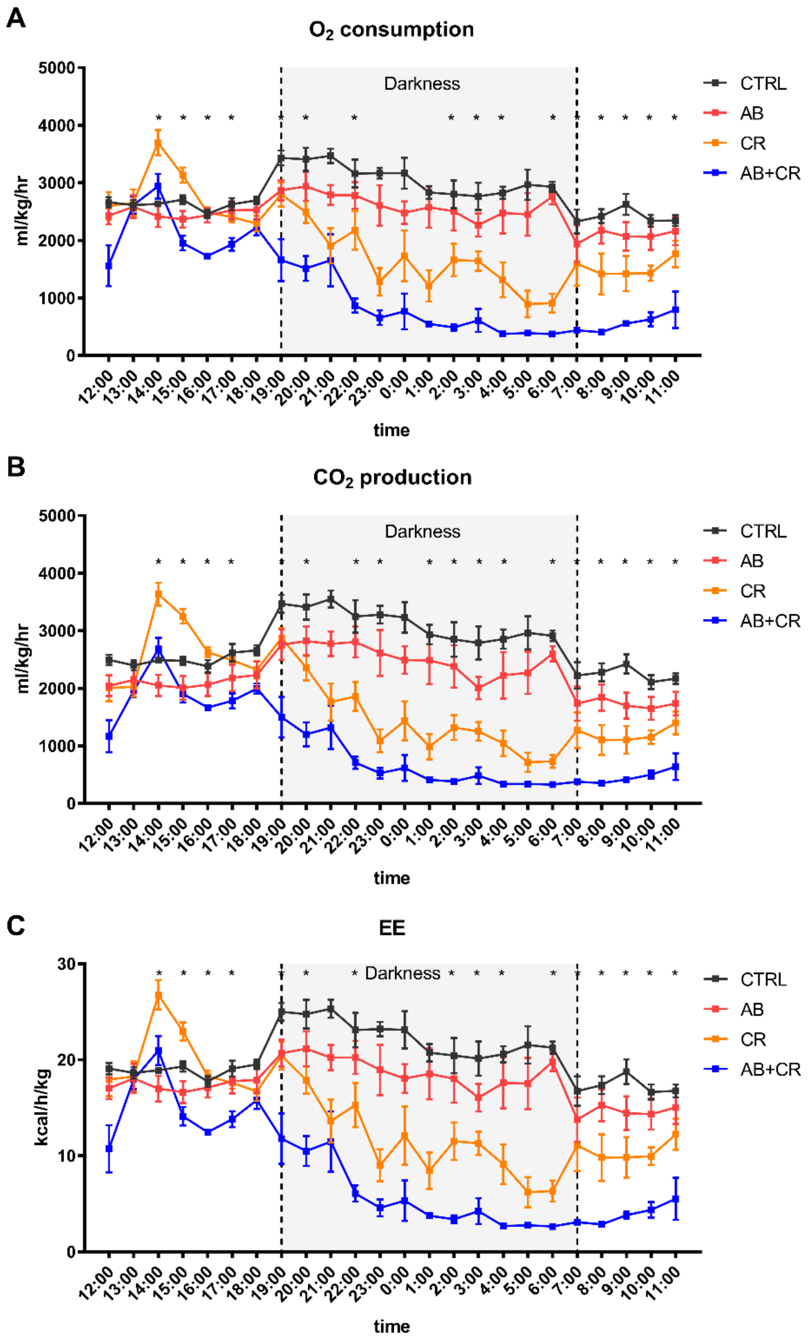

Figure S1. Metabolic indexes as determined by metabolic cages

(A, B) Recordings of O_2 consumption (A) and CO_2 production (B). (C) Analysis of energy expenditure (EE) adjusted to body weight. The asterisks indicate significant differences between CR and AB+CR group. Data are expressed as means ± s.e.m. n = 4 per group. *p < 0.05.

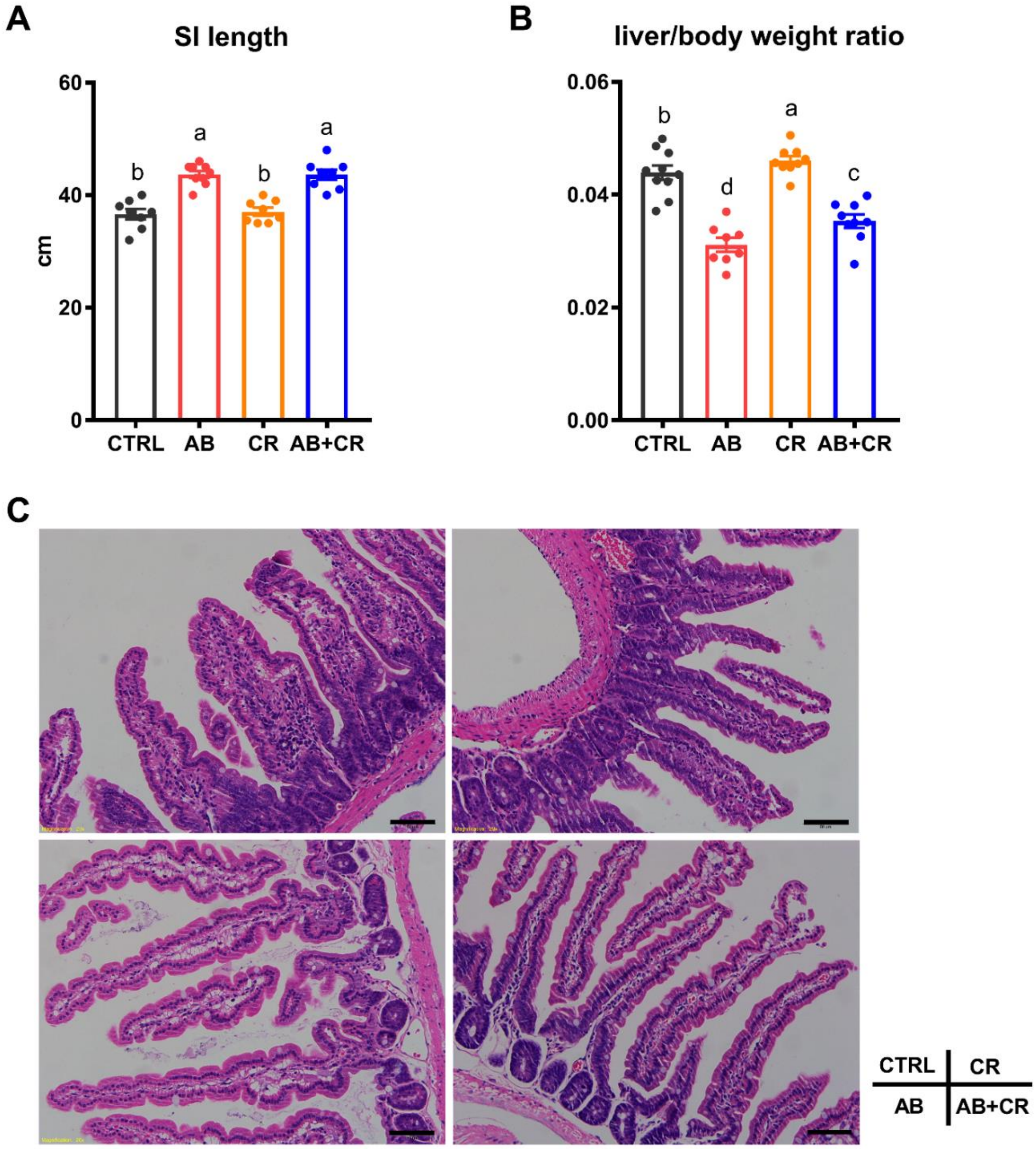

Figure S2. Histological changes of the mice upon CR and antibiotic treatment
(A, B) Differences in the length of small intestine (A) and liver/weight mass ratio (B) in different groups. Data are expressed as means ± s.e.m, n = 8-10 per group. (C) H&E staining of jejunum. Scale bar, 50 μm.

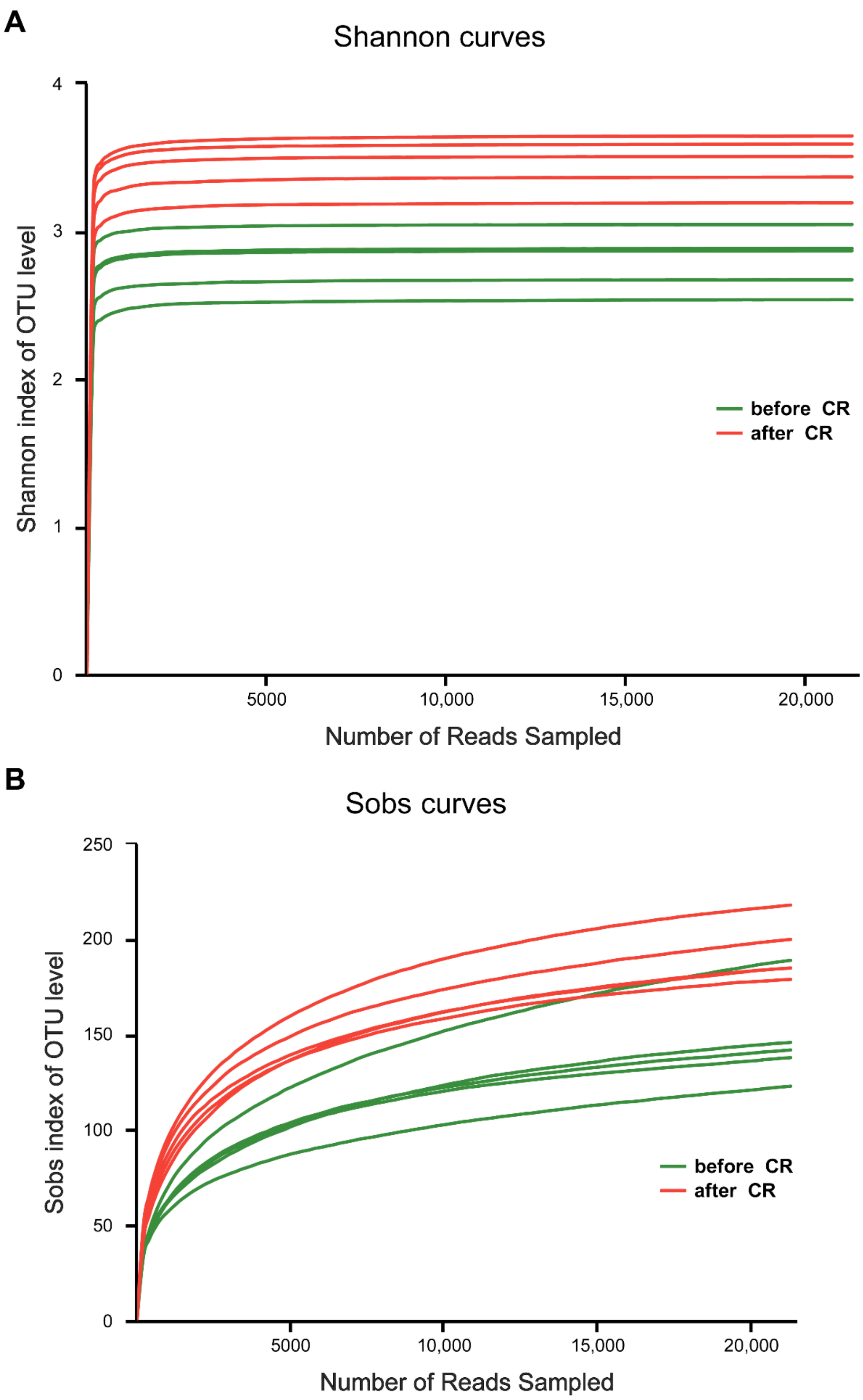

Figure S3. Rarefraction curves of shannon index and sobs index of gut microbes as response to CR

(A, B) Rarefraction curves of shannon index (A) and sobs index (B) of gut microbes as response to CR.

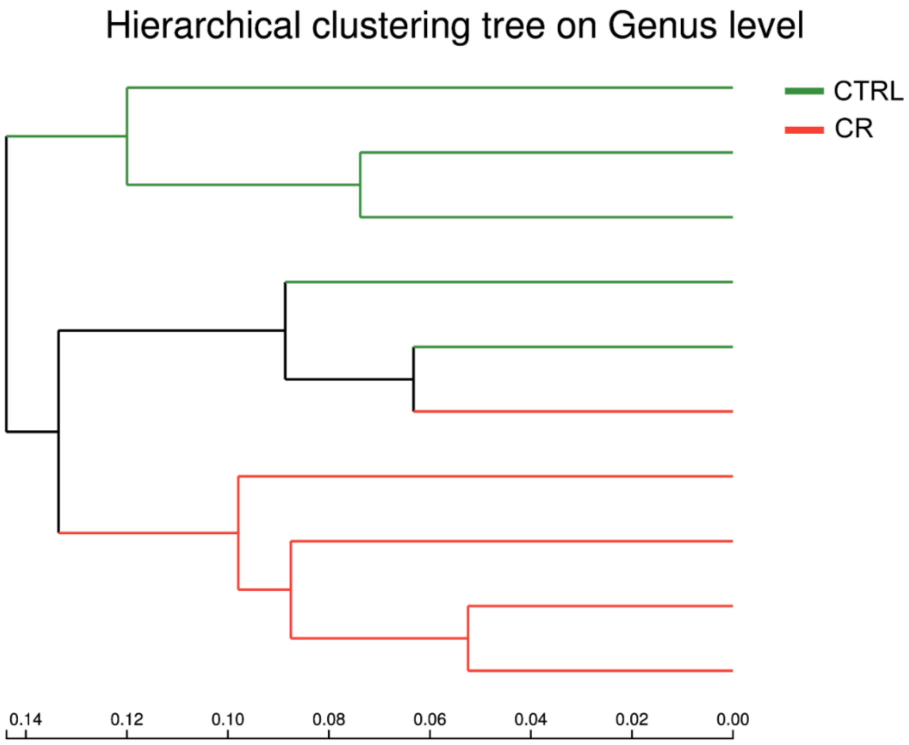

Figure S4. Hierachical clustering of microbes during CR on genus level
Hierachical clustering of microbes during CR on genus level.

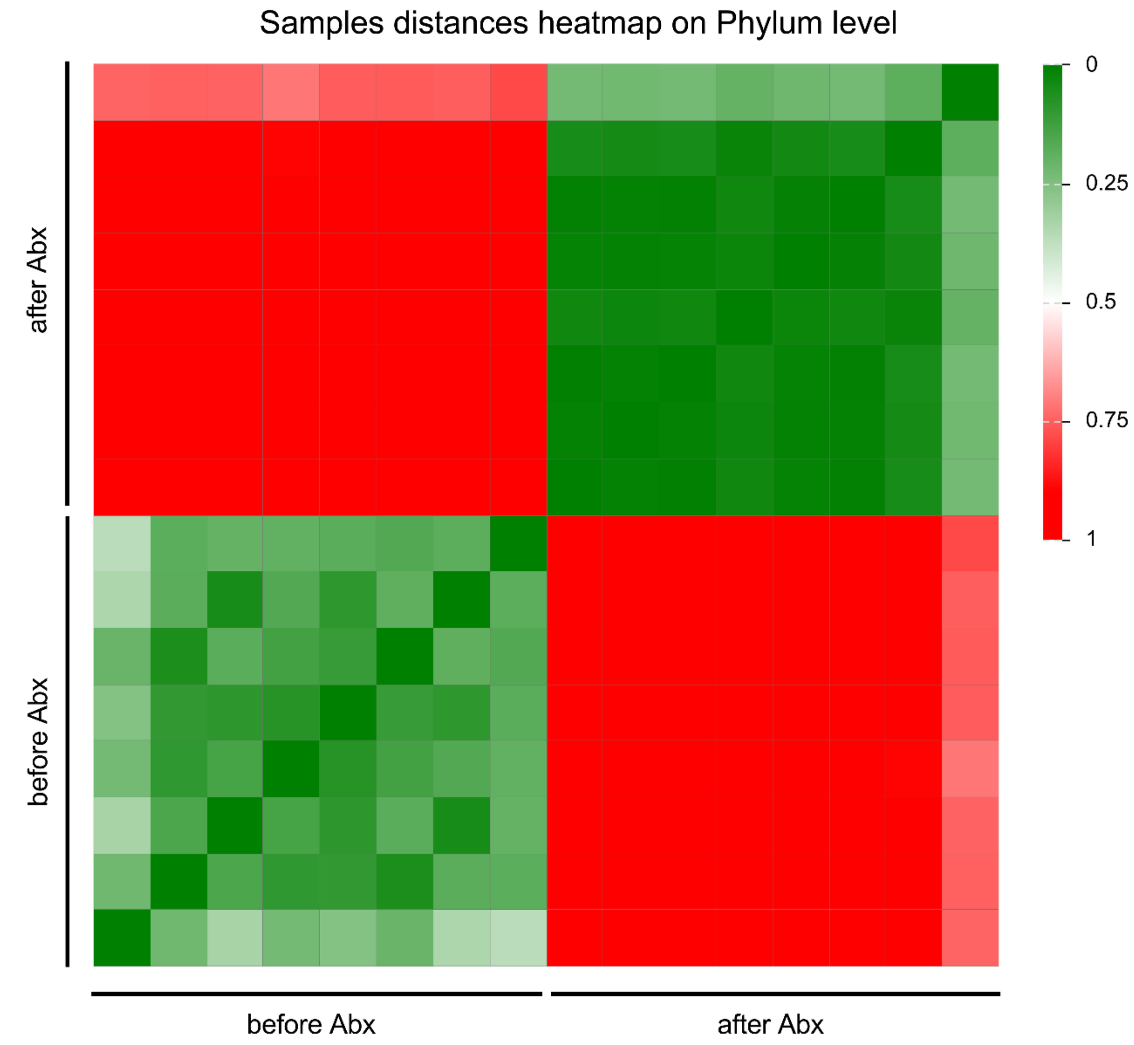

Figure S5. Antibiotic-induced alteration of sample distances on phylum level
Antibiotic-induced alteration of sample distances on phylum level shown in heatmap.

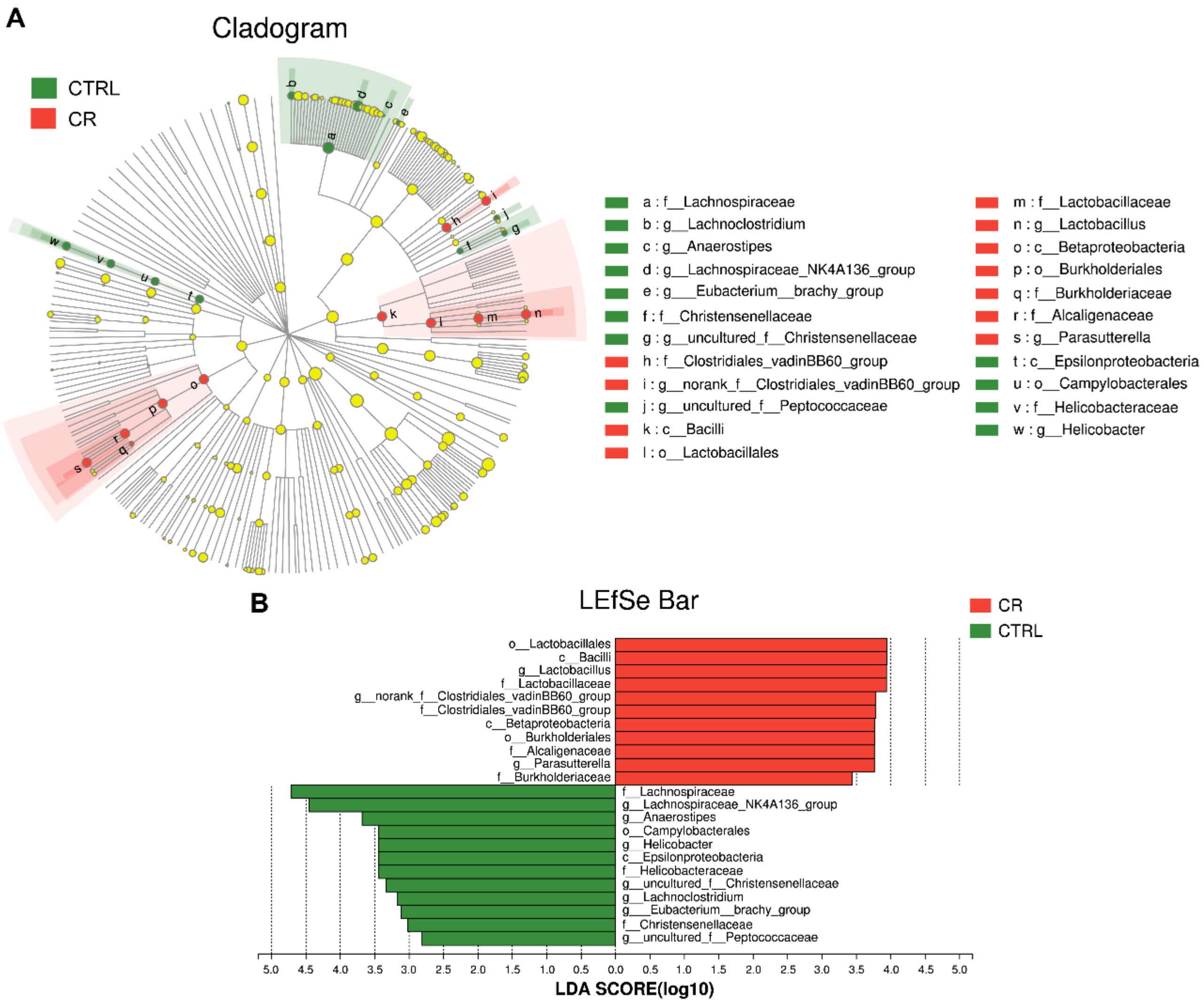

Figure S6. LEfSe analysis of microbial community composition after CR

(A, B) Linear discriminant analysis (LDA) effect size (LEfSe) analysis (A) and corresponding LDA scores (B) in the CTRL and CR groups.

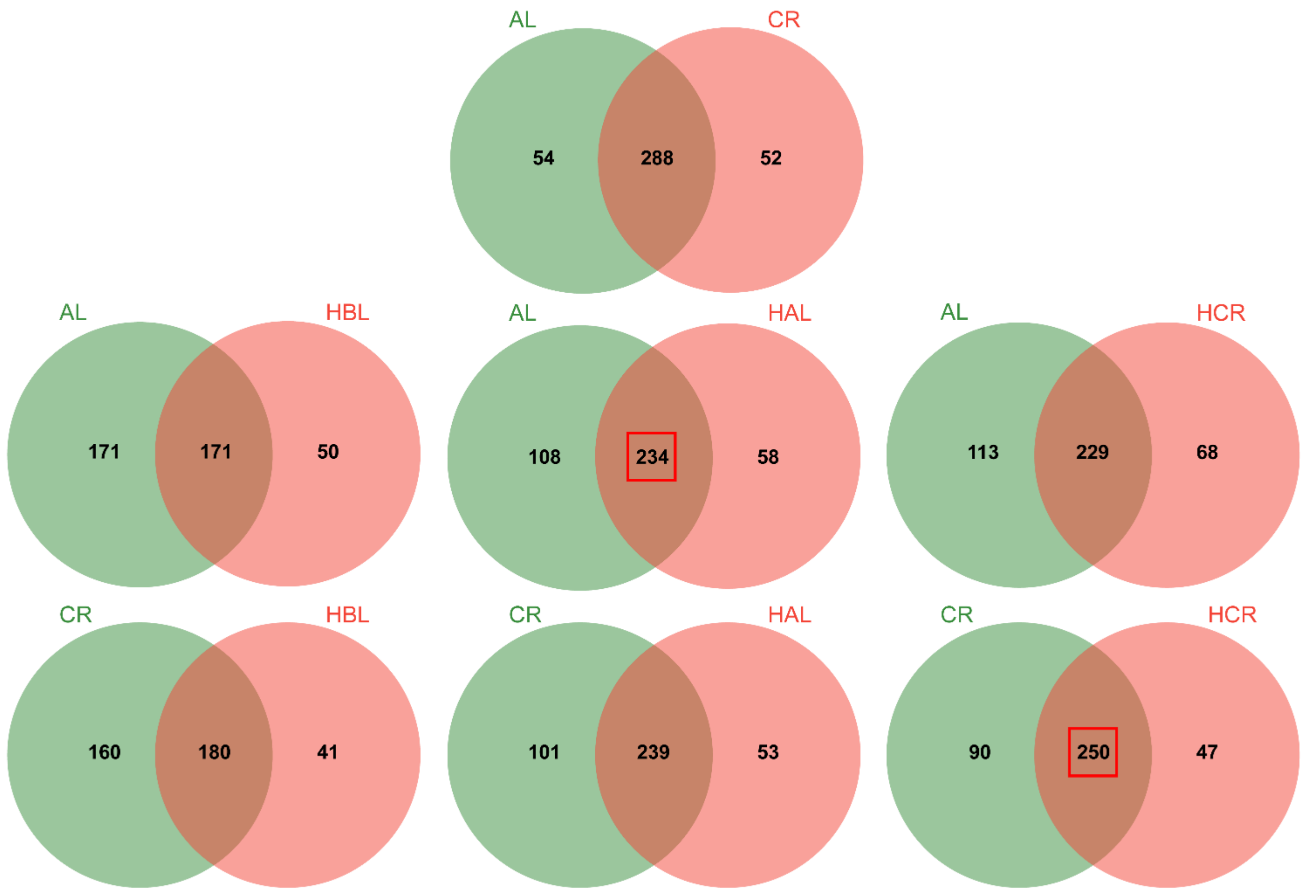

Figure S7. Venn diagrams of microbial community composition at OTU level

Venn diagrams of microbial community composition of corresponding groups at OTU level.

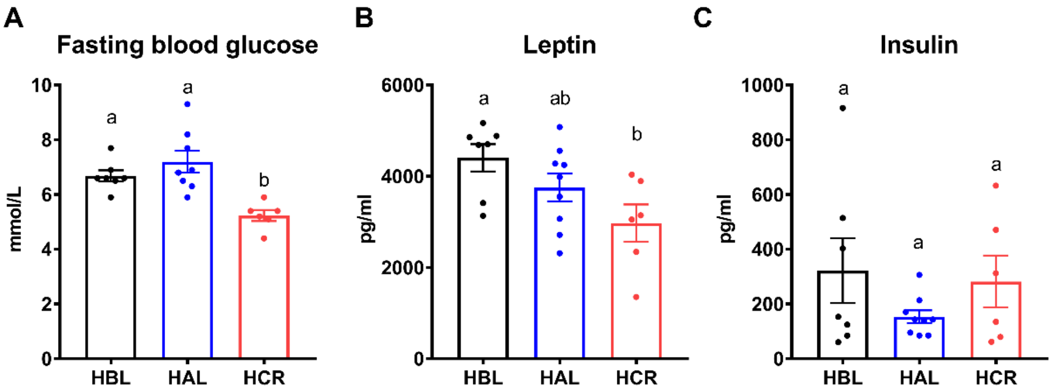

Figure S8. Plasma levels of fasting blood glucose and metabolic hormones after FMT (A-C) Fasting blood glucose (A), plasma leptin (B), and plasma insulin (C) of the mice fed HFD. Data are expressed as means ± s.e.m, n = 6-9 per group.

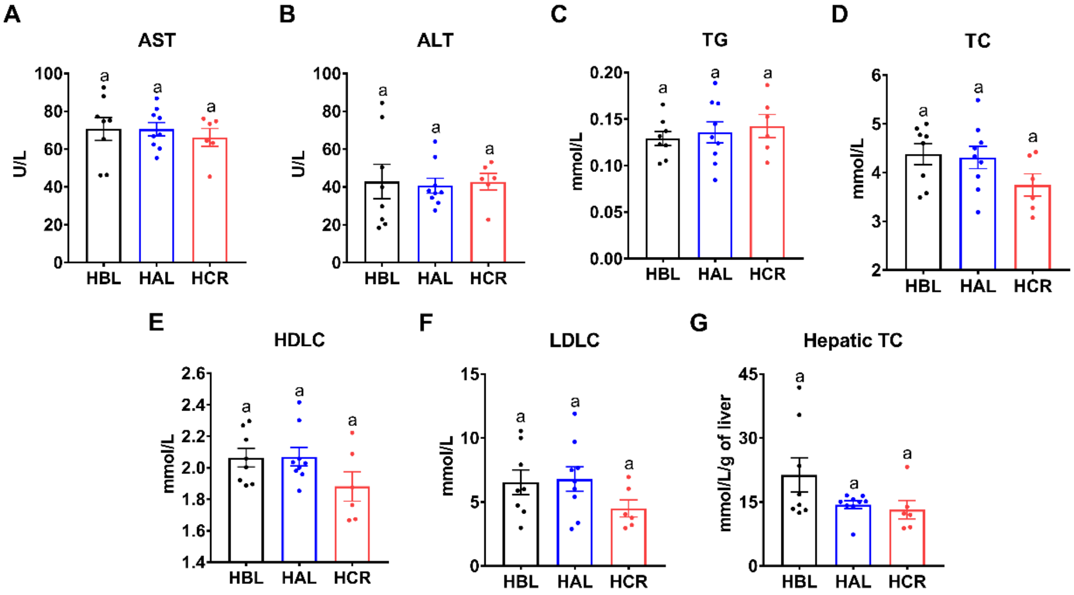

Figure S9. Blood and hepatic parameters after FMT

(A-F) Blood levels of AST (A), ALT (B), TG (C), TC (D), HDL-C (E), and LDL-C (F) of the mice fed HFD.

(G) Hepatic TC level in different groups.

Data are expressed as means ± s.e.m, n = 6-9 per group.

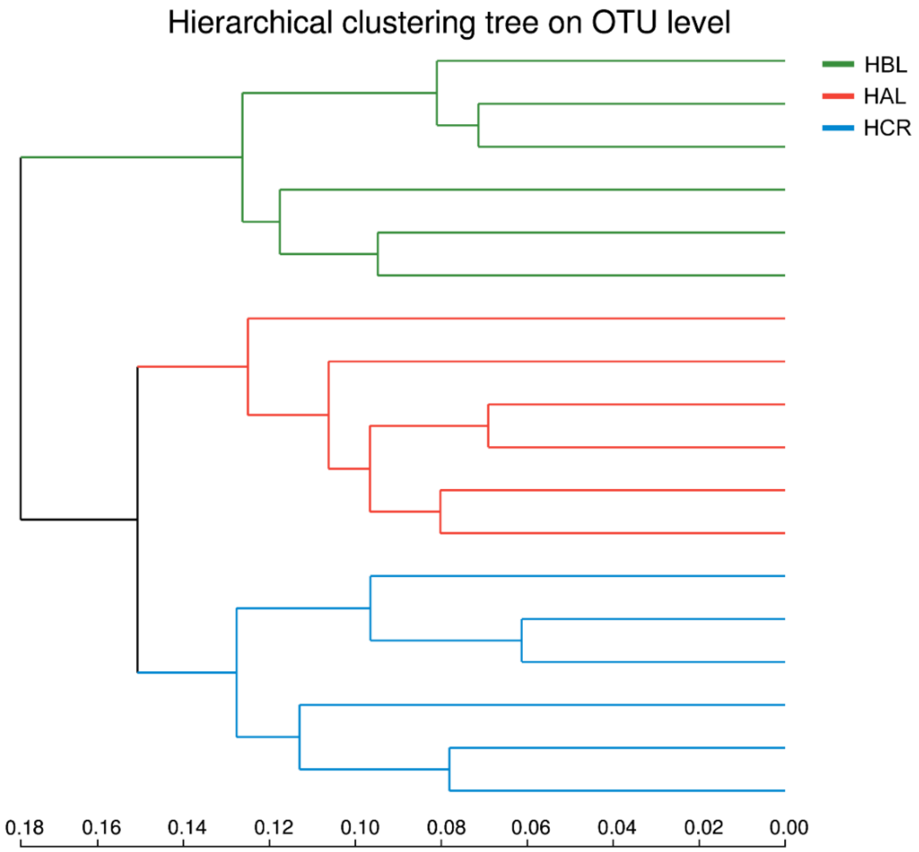

Figure S10. Hierarchical clustering of microbes after FMT on OTU level
Hierarchical clustering of microbes in three groups of mice fed HFD after FMT on OTU level.
